# Characterization of Genetic Diversity in the Capsid Protein Gene of Grapevine Fleck Virus and Development of a New Real-Time RT-PCR Assay

**DOI:** 10.3390/v16091457

**Published:** 2024-09-13

**Authors:** Juliana Osse de Souza, Vicki Klaassen, Kristian Stevens, Teresa M. Erickson, Claire Heinitz, Maher Al Rwahnih

**Affiliations:** 1 Department of Plant Pathology, University of California-Davis, Davis, CA 95616, USA; josouza@ucdavis.edu; 2 Foundation Plant Services, University of California-Davis, Davis, CA 95616, USAtmerickson@ucdavis.edu (T.M.E.); 3 Department of Computer Science, University of California-Davis, Davis, CA 95616, USA; 4 National Clonal Germplasm Repository, Davis, CA 95616, USA; 5 Department of Plant Protection, School of Agriculture, The University of Jordan, Amman 11942, Jordan

**Keywords:** grapevine, grapevine fleck virus, high throughput sequencing, capsid protein phylogeny, RT-qPCR assay design

## Abstract

The grapevine fleck virus (GFkV) is a ubiquitous grapevine-infecting virus found worldwide, is associated with the grapevine fleck complex, and is often found in mixed infections with viruses of the grapevine leafroll complex and/or vitiviruses. Although GFkV has been studied for a long time, limited sequence information is available in the public databases. In this study, the GFkV sequence data available in GenBank and data generated at the Foundation Plant Services, University of California, Davis, were used to perform nucleotide sequence comparisons, construct a phylogenetic tree, and develop a new RT-qPCR assay. Sequence comparisons showed high genetic diversity among the GFkV isolates, and the phylogenetic analyses revealed a new group comprised of GFkV isolates identified in the present study. A new assay, referred to as GFkV-CP, was designed and validated using an existing GFkV positive control together with 11 samples known to be infected with combinations of different marafiviruses and maculaviruses but not GFkV. In addition, the newly designed assay was used in a field survey to screen grapevines from diverse geographical locations that are maintained at the United States Department of Agriculture (USDA) National Clonal Germplasm Repository (NCGR) in Winters, CA.

## 1. Introduction

Grapevines are one of the oldest crops in the history of humanity, with major economic significance in the temperate climate zones around the globe [1], and they are exposed to many different pests and pathogens [2]. There are, to date, more than 100 viruses that have been described associated with grapevine (*Vitis* spp.) worldwide [3]. The grapevine fleck complex is distributed worldwide and is associated with viruses in the family *Tymoviridae* that are genetically closely related but serologically distinct. The grapevine fleck virus (GFkV) member of the species *Maculavirus vitis* and the tentative species grapevine Red Globe virus (GRGV) are very closely related and belong to the genus *Maculavirus*; whereas the grapevine asteroid mosaic associated virus (GAMaV), member of the species *Marafivirus asteroids*;, and grapevine Syrah virus 1 (GSyV-1), member of the species *Marafivirus syrahense*, are members of the genus *Marafivirus*, and the grapevine rupestris vein feathering virus (GRVFV) is a tentative member of this genus.

Although they belong to different genera, these viruses have the following shared features: (i) positive single-stranded RNA genome; (ii) open reading frame (ORF) 1 (in the 5′- end of the RNA) encodes a large polyprotein with signature motifs of several domains associated with viral replication, e.g., methyltransferase, papain-like protease, helicase, and RNA-dependent RNA-polymerase; (iii) ORF 2, downstream of ORF 1, encodes for the capsid protein (CP); (iv) the virions are isometric and non-enveloped; (v) are graft-transmissible; and (vi) no known insect vector [4,5].

For a long time, registration and certification programs have relied on biological indexing onto woody plants and mechanical inoculation onto herbaceous hosts for the testing and releasing of new registered/certified clean grapevine propagative material [6]. GFkV causes fleck symptoms on the indicator *V. rupestris* and is therefore part of the registration and certification protocols all around the world. However, there is no evidence of disease symptoms associated with GFkV in any other *Vitis* spp. [4]. Furthermore, the impact of GFkV on vine vigor and fruit yield and quality is negligible [7].

The California Grapevine Registration and Certification (R&C) Program is administered by the California Department of Food and Agriculture (CDFA), and it targets the elimination of specific grapevine diseases. Under the CDFA program, grapevine materials are introduced, disease tested and then identified to become part of the foundation vineyard blocks. Foundation Plant Services (FPS) at the University of California, Davis (https://fps.ucdavis.edu/, accessed on 1 August 2024), is responsible for the registration and maintenance testing of grapevine foundation collections in the R&C program. Hence, through the pipeline of domestic and foreign introductions, FPS produces virus-tested grapevine material, maintains the clonally propagated foundation vineyard, and is the source of all California registered or certified grapevines. To maintain and produce virus-tested and clonally propagated foundation stock, reliable and diagnostic methods are imperative.

In 2023, nine of the plants in the FPS pipeline of the domestic and foreign introductions annual testing produced questionable signals, suggesting low specificity (cycle quantification [Cq] values > 30) in the RT-qPCR tests using the GFkV-Rep assay [8]. The high throughput sequencing (HTS) results of these plants indicated that they were positive for GSyV-1 but negative for GFkV. This suggested that the GFkV-Rep assay was not specific to GFkV but could cross-react with other closely related viruses in the family *Tymoviridae*. The purpose of this work was to develop a new sensitive and specific GFkV RT-qPCR assay.

## 2. Materials and Methods

### 2.1. Plant Material and Virus Source

FPS, as a clean plant center, provides services of introductions, testing, and virus elimination for grapevine material from all around the world. Every year, the introduced plants go through the HTS pipeline, and the data generated make up the FPS internal database of metagenomes. Samples positive for GFkV were obtained from the pipeline of domestic and foreign introductions and were used in the present study (Supplementary Table S1).

### 2.2. High Throughput Sequencing

For the FPS pipeline of foreign and domestic introductions, a total of 700 ng per 10 µL of extracted nucleic acid was subjected to rRNA depletion and cDNA library construction using the TruSeq Stranded Total RNA with Ribo-Zero Plant Kit (Illumina, San Diego, CA, USA). Subsequently, the cDNA was end-repaired, adapter ligated by unique dual indexes, and then PCR enriched. Library quality was assessed using a Bioanalyzer 2100 (Agilent, Santa Clara, CA, USA). Finally, the amplicons were sequenced in an Illumina NextSeq 500 platform using single-end 75 bp reads. The Illumina reads went through the bioinformatics pipeline previously described [9]. Briefly, the reads were demultiplexed using bcl2fastq2 and subsequently assembled using spades [10]. Assembled contigs were identified as GFkV using blastn and blastx searches against the GenBank nt and nr databases, respectively.

### 2.3. Sequence and Phylogenetic Analyses

Pairwise sequence comparisons were performed with MUSCLE within the Sequence Demarcation Tool (SDT) v.1.2 [11]. Amino acid and nucleotide sequence alignments and comparisons were performed with MUSCLE v.3.5 within the Geneious Prime 2024.0.5 software (GraphPad Software LLC d.b.a. Geneious, Boston, MA, USA). The best-fit model of nucleotide substitution was determined with the program ModelTest-NG [12].

The phylogenetic analysis was performed, and trees were constructed using Bayesian inference performed with BEAST v.2.5 [13]. For this analysis, a constant population size was assumed, with a log-normal relaxed molecular clock. The Markov chain Monte Carlo (MCMC) simulation was run for 100,000,000 generations and sampled at 10,000 trees. The maximum clade credibility trees were made by using Tree-Annotator v.2.5.1, discarding the first 25% of the MCMC chains as burn-in with a posterior probability (pp) limit of 0.5 and with highest posterior probabilities (HPD) of 95%. Trees were edited in FigTree v.1.4.4 (http://tree.bio.ed.ac.uk/software/figtree/, accessed on 22 May 2024).

### 2.4. Development of a New GFkV RT-qPCR Assay

For the design of the new assay, first, MUSCLE [14] was used to evaluate the conservation and depth of the sequence information present in the capsid protein (CP) ORF of all the virus sequences deposited in GenBank together with sequences from the FPS internal database of metagenomes. This information was then used for optimal minor grove binder (MGB) probe placement, followed by forward and reverse primer placement within 100 bp to the left and right of the probe. Primer Express software (ThermoFisher Scientific Inc., Foster City, CA, USA) was used to optimize final primer and probe sequences according to the parameters for RT-qPCR with MGB probes. Primer–primer and primer–probe interactions were also evaluated using the same software.

### 2.5. RT-qPCR Assay Validation

Assay sensitivity and specificity were tested using the following three different sets of samples: (i) a known GFkV positive control (used to generate the standard curve) from the Davis Virus Collection (DVC) at University of California, Davis [15]; (ii) nine original samples in which the low specificity was identified; and (iii) 11 grapevines infected with different combinations of GAMaV, GRGV, GRVFV, and GSyV-1 but not GFkV (Table 1).

The above-mentioned samples were subjected to total nucleic acid extraction as follows: First, 0.15 g of plant tissue was homogenized in 3 mL of guanidine isothiocyanate lysis buffer (4 M guanidine isothiocyanate; 0.2 M sodium acetate, pH 5.0; 2 mM EDTA; 2.5% (*w*/*v*) PVP-40) and the TNA extracts were then prepared using a MagMax^TM^ viral RNA isolation kit (ThermoFisher Scientific Inc., Foster City, CA, USA) following the manufacturer’s protocol.

RT-qPCR reactions were completed in the QuantStudio 6 real-time PCR system using the TaqMan Fast Virus 1-Step Master Mix and following the recommended protocol. Each reaction (10 µL final volume) included 2 µL of RNA and final primer and probe concentration of 900 and 250 ƞM, respectively [16]. The thermocycler conditions were as follows: 50 °C for 5 min, 95 °C for 20 s, followed by 40 cycles of 95 °C for 3 s, and 60 °C for 30 s. Additionally, the assay was multiplexed with a previously published 18S rRNA assay [17] to verify the presence of high-quality RNA during the reaction.

The efficiency and sensitivity of the RT-qPCR assay was determined using serial dilutions (1:1 to 1:1,000,000) of RNA extracts in water and run in triplicate. Standard curves were calculated using the QuantStudio 6 real-time PCR software [18].

### 2.6. Large-Scale Testing Using the New Assay

The National Clonal Germplasm Repository (NCGR), a United States Department of Agriculture genetic resource, is located near Winters, California, and contains approximately 4500 grapevines representing different accessions. Plants in this collection originated globally (https://www.ars.usda.gov/pacific-west-area/davis-ca/natl-clonal-germplasm-rep-tree-fruit-nut-crops-grapes/, accessed on 1 August 2024). In a previous study [18], the grapevines known to be infected by grapevine leafroll-associated viruses were sampled and the RNA extracted. For the present study, 1102 accessions of those used by Diaz-Lara et al. [18] were tested by RT-qPCR.

## 3. Results

### 3.1. New Genetic Diversity among GFkV Isolates

To design a new GFkV RT-qPCR assay for increased specificity, we analyzed the GFkV nucleotide sequence data from both GenBank and the FPS internal database of metagenomes. While the GenBank data for GFkV were limited, the FPS internal database included genetically diverse GFkV isolates from different parts of the world. Although no nearly complete genomic sequences were found, 73 new GFkV nucleotide sequences encoding a complete copy of the CP were identified and deposited in GenBank (accession numbers: PP481473 to PP481549).

Pairwise nucleotide sequence comparisons were next performed with the complete nucleotide sequence of the CP ORF with the 73 new GFkV isolates and 53 isolates retrieved from the GenBank, for a total of 126 GFkV isolates. The heat map of the Sequence Demarcation Tool (SDT) analysis with the CP comparisons revealed identities ranging from 84.3 to 99.9% (Supplementary Figure S1), indicating that these are isolates of GFkV that represent a high degree of genetic diversity. The sequence of each isolate had at least one value equal to or greater than the ICTV-recognized species demarcation threshold of one capsid protein sequence identity > 90% to another GFkV sequence [19].

**Figure 1:**
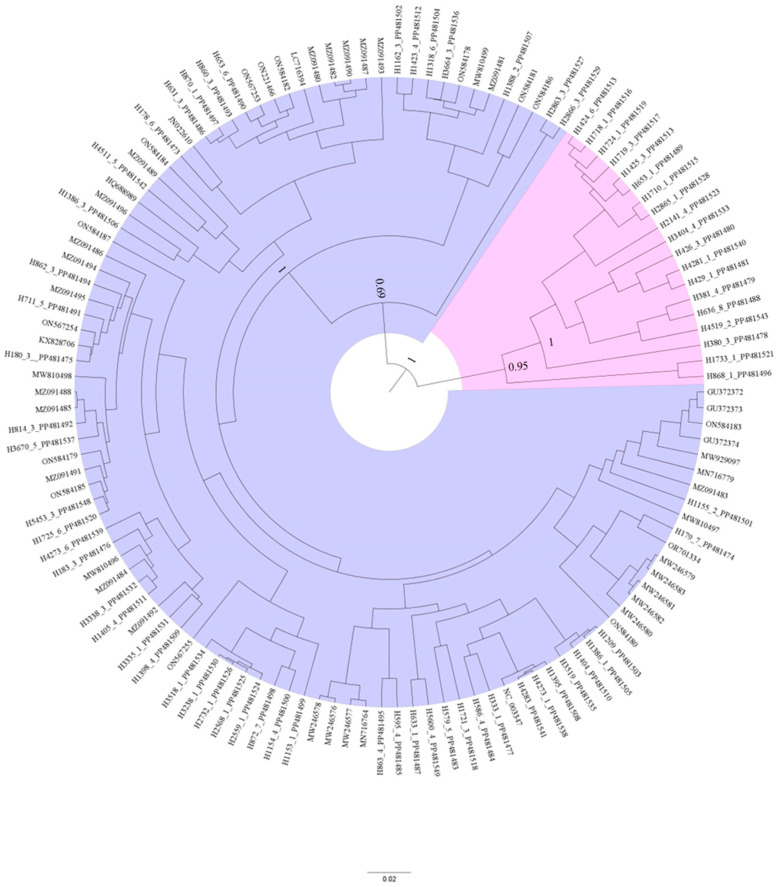
Bayesian phylogenetic consensus tree generated from an alignment of the complete open reading frame (ORF) 2, which encodes the capsid protein (CP), of 73 grapevine fleck virus (GFkV) isolates generated in the present study and 53 GFkV isolates from GenBank, for a total of 126 sequences. The phylogenetic analysis was performed with BEAST v.2.5. Branch strengths were evaluated by Bayesian posterior probabilities; posterior probabilities for the two most important clades are shown. The length of horizontal branches corresponds to the rate of nucleotide substitution. GenBank accession numbers are given.

To understand the high degree of genetic diversity and the relationship among the GFkV isolates an unrooted phylogenetic tree was constructed. The tree shows the evolutionary relationships presented in Figure 1 and comprises the 126 nucleotide sequences of the CP ORF of GFkV isolates. These isolates were placed into two strongly supported clades (pink and lilac). The lilac clade corresponds to the previously characterized GFkV isolates retrieved from the GenBank together with some of the GFkV isolates identified from the internal FPS metagenomes database. The other strongly supported clade (pink) includes only the GFkV isolates from this study. These results suggest that these isolates from the present study constitute a new lineage of GFkV isolates.

### 3.2. Assay Design and Validation

**Figure 2:**
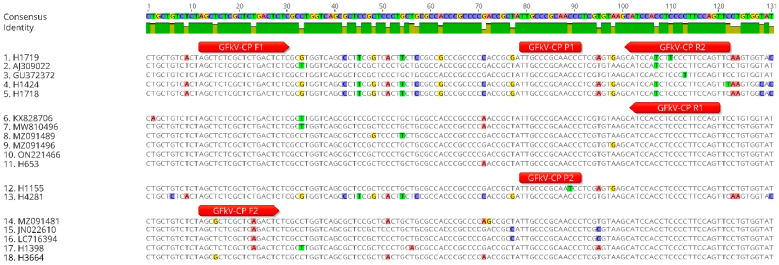
Alignment of partial capsid protein gene of selected grapevine fleck virus (GFkV) isolates showing the binding sites of the primers and probes developed in this study. The red horizontal bars represent the primers and probes of the GFkV-CP assay designed in this study, the sequences of primers and probes are directly below the bars. Nucleotide divergence is highlighted in different colors for each nucleotide.

To avoid the non-specific amplification observed with the GFkV-Rep RT-qPCR assay, and to accommodate the new genetic diversity described in the CP ORF, we designed a new GFkV assay to amplify a region at the 5′ end of the CP that was conserved within the 126 GFkV isolates but divergent among marafiviruses and other maculaviruses. The new assay, GFkV-CP, includes two forward and two reverse primers and two MGB probes (Table 2 and Figure 2).

For the first level of assay validation, a standard curve was generated using the positive control collected from the DVC. The slope of the standard curve (−3.447) was used to calculate the amplification efficiency of 95.036% with a coefficient of correlation (R2) of 0.99 (Supplementary Figure S2). For the second level of validation, the 9 original samples that generated high Cq values and the 11 samples with mixed infections of closely related viruses but not GFkV were tested. The RT-qPCR test showed no amplification for the above-mentioned samples when the new assay was used whereas the positive control showed amplification (Cq = 17) and were amplified by their respective assay. These results show GFkV-CP assay has higher specificity in comparison to GFkV-Rep.

### 3.3. Large-Scale Testing by GFkV-CP Assay

Lastly, we used the new GFkV-CP assay to evaluate the 1102 grapevine accessions collected previously for a survey on grapevine leafroll-associated viruses [18]. These accessions originated from the USDA NCGR grapevine germplasm collection. To this end, GFkV was detected in 189 out of 1102, or 17.1% of the accessions tested (Table 3), with Cq values ranging from 14 to 31 and a mean of 22. The positive grapevine samples originated from 21 countries (Table 3).

## 4. Discussion

To avoid the low specificity observed with the GFkV-Rep RT-qPCR assay, we designed a new GFkV assay by identifying a conserved region on the GFkV genome that would not cross-react with GRGV or marafiviruses. Because the GFkV sequence availability in the GenBank was limited, we searched the FPS internal database of viral metagenomes for GFkV sequences and identified 73 new complete nucleotide sequences of the GFkV CP ORF.

Sequence comparisons performed using the complete CP ORF from the 73 new sequences together with the 53 sequences available in the GenBank showed the previously uncharacterized genetic diversity in the CP gene. Previous phylogenetic studies of GFkV have been mostly performed with partial sequences of the replicase ORF [20,21,22,23,24] rather than complete sequences of the CP ORF. In addition, comparisons have been made among members of the family Tymoviridae not within the GFkV species. Glasa et al., 2011 [25], performed comparisons with the partial CP ORF among the isolates of GFkV, and they also noted two distinct phylogenetic groups. This genetic diversity is evenly distributed on the CP gene and may not affect the virus replication cycle.

Similar to the phylogenies of GFkV, most of the published assays target a conserved region of the replicase ORF [4,8,26]. For the GFkV-CP assay, a conserved region on the 5′ end of the CP gene was identified, and primers and probes were designed in this location of the genome. The primers and probes designed for this assay are multiplex and detect the known variants of the virus, which is essential to reduce the risk of false negative test results because of nucleotide mismatch.

The GFkV RT-qPCR assays were first tested side-by-side using the original samples in which the low specificity was identified. The GFkV-Rep assay continued to generate Cq > 30 while the GFkV-CP assay results were negative, confirming the higher specificity of the new GFkV-CP assay.

To validate the GFkV-CP assay, a two-step strategy was employed. For the first step, RNA extracts prepared from 11 grapevines infected with the closely related viruses, GRGV, GRVFV, GSyV-1, and GAMaV were tested. No amplification occurred, indicating that the assay was specific for GFkV. For the second step, RNA extracts were prepared from grapevine samples originating from 46 different countries across five continents. This large-scale survey on the NCGR samples provided evidence that the GFkV-CP assay is reliable when tested on samples from a genetically diverse germplasm and from different parts of the world. The extensive validation presented here indicates that the GFkV-CP has high diagnostic sensitivity.

RNA viruses have a high mutation rate and, as a consequence, extensive genetic diversity [27]. Although GFkV is widespread in the world, the genetic diversity of this virus is underrepresented, with fewer than 10 complete sequences in the GenBank. To address this gap and still provide the necessary nucleotide sequence data required for RT-qPCR assay design, we used not only sequences from the GenBank but also added a number from our internal metagenomes database. The new sequences increase our understanding of the genetic diversity within the species, including revealing a new divergent clade and allowing for the development of the new sensitive and specific GFkV-CP assay.

## Figures and Tables

**Table 1:** Grapevine samples obtained from the Foundation Plant Services (FPS, University of California, Davis) pipeline of domestic and foreign introductions and known to be infected by viruses closely related grapevine fleck virus (GFkV) and used in the specificity assay.

Sample Designation	Viruses Present in the Sample ^a^
1	GRGV, GRVFV, GSyV-1
2	GRGV, GRVFV, GSyV-1
3	GRGV, GRVFV, GSyV-1
4	GRGV, GRVFV, GSyV-1
5	GRGV, GRVFV, GSyV-1
6	GRGV, GRVFV, GSyV-1
7	GRGV, GRVFV, GSyV-1
8	GRGV, GSyV-1
9	GRGV, GRVFV
10	GAMaV, GSyV-1
11	GRGV, GSyV-1

^a^ Acronym of the virus species name proven to be present in the samples by high throughput sequencing. GRGV: Grapevine Red Globe virus, GRVFV: grapevine rupestris vein feathering virus, GSyV-1: grapevine Syrah virus 1 and, GAMaV: grapevine asteroid mosaic-associated virus.

**Table 2:** The new grapevine fleck virus capsid protein (GFkV-CP) assay for detection of grapevine fleck virus.

Virus	Oligo Name	Sequence (5′ to 3′)	5′ Reporter	Probe Type	Target Region
GFkV	GFkV-F1	AGCTCTCGCTCTGACTCTC			CP
	GFkV-R1	ACTGGAAGGGGAGGTGGAT			
	GFkV-F2	AGCGCTCGCTCAGACTC			
	GFkV-R2	GAACTGGAAGGGAAGATGGATG			
	GFkV-P1	TTGCCCGCAACCC	FAM	MGB	
	GFkV-P2	TTGCCCGCAATCC	FAM	MGB	

**Table 3:** Detection of grapevine fleck virus by GFkV-CP assay in the National Clonal Germplasm Repository in grapevine samples from diverse origin.

Country of Origin	Positive Samples
Afghanistan	3
Australia	2
Austria	9
Canada	6
China	1
France	8
Germany	3
Greece	4
Italy	17
Japan	2
Morocco	1
Pakistan	7
Portugal	12
Russian Federation	4
Serbia	2
South Africa	4
Soviet Union	3
Spain	1
Ukraine	1
United Kingdom	1
United States	98

## Data Availability

Grapevine fleck virus capsid protein gene nucleotide sequences are available at NCBI GenBank with the following accession numbers: H178_6, PP481473; H179_7, PP481474; H180_3, PP481475; H183_3, PP481476; H333_1, PP481477; H380_3, PP481478; H381_4, PP481479; H426_3, PP481480; H429_1, PP481481; H450_1, PP481482; H579_5, PP481483; H586_4, PP481484; H595_4, PP481485; H631_3, PP481486; H633_1, PP481487; H636_8, PP481488; H653_1, PP481489; H653_6, PP481490; H711_5, PP481491; H814_3, PP481492; H860_3, PP481493; H862_3, PP481494; H863_4, PP481495; H868_1, PP481496; H870_1, PP481497; H872_7, PP481498; H1153_1, PP481499; H1154_4, PP481500; H1155_2, PP481501; H1162_3, PP481502; H1209, PP481503; H1318_6, PP481504; H1386_1, PP481505; H1386_3, PP481506; H1388_2, PP481507; H1395, PP481508; H1398_4, PP481509; H1404, PP481510; H1405_4, PP481511; H1423_4, PP481512; H1424_6, PP481513; H1425_3, PP481514; H1710_1, PP481515; H1718_1, PP481516; H1719_3, PP481517; H1721_3, PP481518; H1724_1, PP481519; H1725_6, PP481520; H1733_1, PP481521; H1954_1, PP481522; H2141_4, PP481523; H2559_1, PP481524; H2568_1, PP481525; H2732_1, PP481526; H2863_3, PP481527; H2865_1, PP481528; H2866_3, PP481529; H3238_1, PP481530; H3335_1, PP481531; H3338_3, PP481532; H3404_4, PP481533; H3518_1, PP481534; H3519, PP481535; H3664_3, PP481536; H3670_5, PP481537; H4273_1, PP481538; H4273_6, PP481539; H4281_1, PP481540; H4283, PP481541; H4511_5, PP481542; H4519_2, PP481543; H5167_3, PP481547; H5453_3, PP481548; H5600_4, PP481549.

## Supplementary Materials

The following supporting information can be downloaded at: https://www.mdpi.com/article/10.3390/v16091457/s1, Figure S1: Sequence Demarcation Tool generated heat map showing the pairwise nucleotide identities of the complete open reading frame (ORF) 2, which encodes the capsid protein (CP), of 73 Grapevine fleck virus (GFkV) isolates generated in the present study and 53 GFkV isolates from GenBank, for a total of 126 sequences; Figure S2: Relative grapevine fleck virus (GFkV) quantification of the GFkV-CP assay. (**a**) Amplification plot and (**b**) Standard curve. Cycle quantification (Cq) values obtained for three replicates of ten-fold serial dilutions of GFkV control are plotted; Table S1: Origin of the grapevine fleck virus isolates obtained in this study.
