# Supplementary material for: Characterization of Genetic Diversity in the Capsid Protein Gene of Grapevine Fleck Virus and Development of a New Real-Time RT-PCR Assay
Source: Viruses. 2024 Sep 13;16(9):1457. doi: 10.3390/v16091457 (PMC11437500; doi:10.3390/v16091457)
Supplement: Supplementary file 1 [file viruses-16-01457-s001.zip › viruses-3181955-supplementary.pdf]

Supplementary Table S1. Origin of the grapevine fleck virus isolates obtained in this study.

Isolate	Collection Date	Country	Cultivar
H178_6	25-Jan-17	Pakistan	Bai-Ji-Xin
H179_7	25-Jan-17	Pakistan	Italia
H180_3	25-Jan-17	Pakistan	
H183_3	25-Jan-17	Pakistan	Aphonso
H333_1	1-Jun-17	USA	Greco Nero
H380_3	6-Sep-17		
H381_4	6-Sep-17		
H426_3	9-Oct-19	USA	Noir Hatif de Marceille
H429_1	1-Oct-19	USA	Mtsvane Kakhuri
H450_1	5-Jul-18	USA	Merlot 32
H579_5	9-Oct-19	USA	Pinot Noir
H586_4	21-Aug-18	Greece	Assýrtiko
H595_4	21-Aug-18	USA	Grenache Noir
H631_3	21-Aug-18	USA	Grenache Blanc
H633_1	21-Aug-18	USA	Kozma 55
H636_8	21-Aug-18	USA	Petit Bouschet
H653_1	21-Aug-18	Croatia	Vugava
H653_6	21-Aug-18	Croatia	Vugava
H711_5	23-Apr-18	Austria	Harslevelu
H814_3	23-Apr-18	USA	Pinot Noir 1A4
H860_3	5-Jul-18	USA	
H862_3	5-Jul-18	USA	
H863_4	5-Jul-18	USA	
H868_1	5-Jul-18	USA	
H870_1	5-Jul-18	USA	
H872_7	5-Jul-18	USA	
H1153_1	4-Feb-19	USA	Cabernet Sauvignon
H1154_4	4-Feb-19	USA	St. George
H1155_2	4-Feb-19	USA	St. George
H1162_3	4-Feb-19	USA	Super Hamburg
H1209	1-Mar-19	USA	Chardonnay
H1318_6	1-Mar-19	USA	Nagano Purple
H1386_1	27-Mar-19	USA	Sémillon
H1386_3	27-Mar-19	USA	Sémillon
H1388_2	27-Mar-19	USA	Croatina
H1395	2-Apr-19	Greece	Athiri
H1398_4	2-Apr-19	Greece	Sultanina
H1404	2-Apr-19	Croatia	Pošip bijeli
H1405_4	2-Apr-19	France	Grenache Blanc
H1423_4	10-May-19	Georgia	Akhmetis Tsiteli
H1424_6	15-Apr-19	Croatia	Babica
H1425_3	15-Apr-19	Croatia	Dobričić
H1710_1	24-Jul-19	Croatia	Dobričić
H1718_1	24-Jul-19	Croatia	Babic
H1719_3	24-Jul-19	Croatia	Maraština
H1721_3	24-Jul-19	Croatia	Plavac Mali
H1724_1	24-Jul-19	Croatia	Babica
H1725_6	24-Jul-19	Croatia	Pošip
H1733_1	24-Jul-19	Croatia	Pošip

H1954_1	9-Nov-20	USA	Merlot
H2141_4	26-Feb-20	USA	St. George
H2559_1	31-Jul-20	USA	
H2568_1	31-Jul-20	USA	
H2732_1	2-Sep-20	USA	
H2863_3	19-Nov-20	USA	
H2865_1	19-Nov-20	USA	
H2866_3	19-Nov-20	USA	
H3238_1	21-May-21	USA	
H3335_1	10-Aug-21	Israel	Shami
H3338_3	10-Aug-21	China	Voivoginiova
H3404_4	27-Jul-21	USA	St. George
H3518_1	15-Sep-21	USA	
H3519	15-Sep-21	USA	
H3664_3	17-Nov-21	USA	Chardonnay
H3670_5	17-Nov-21	USA	Catarratto
H4273_1	10-Mar-22	Lebanon	Obeidi
H4273_6	10-Mar-22	Lebanon	Obeidi
H4281_1	10-Mar-22	USA	GEM 4
H4283	10-Mar-22	Georgia	Otskhanuri Sapere
H4511_5	17-May-22	France	Petit Meslier
H4519_2	17-May-22	France	Pinot Noir
H5453_3	22-Feb-23	USA	Merlot
H5600_4	11-Apr-23	France	Grolleau

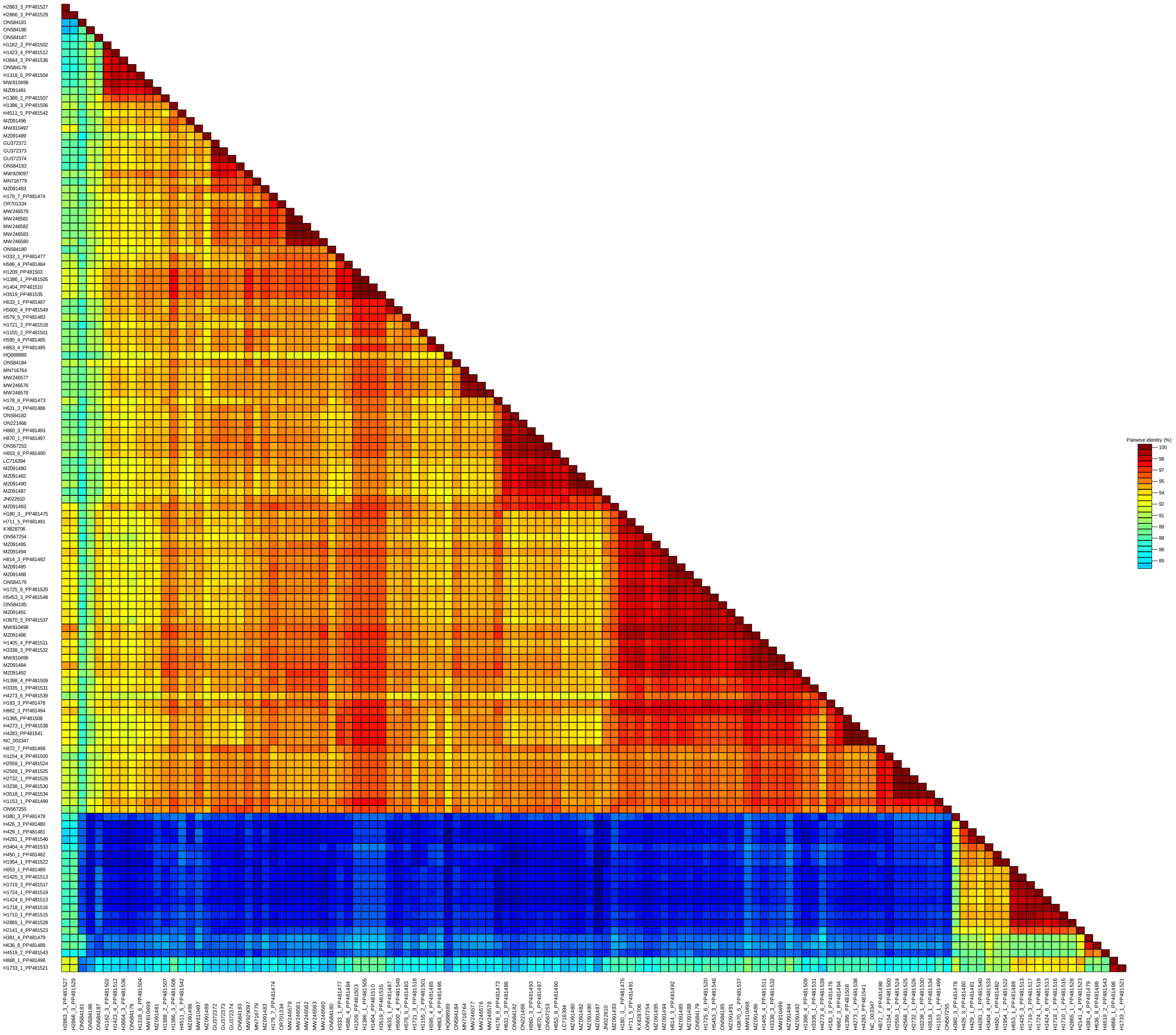

Supplementary Figure S1. Sequence Demarcation Tool generated three colored heat map showing the pairwise nucleotide identities of the complete open reading frame (ORF) 2, which encondes the capsid protein (CP), of 73 Grapevine fleck virus (GFkV) isolates generated in the present study and 53 GFkV isolates from GenBank, for a total of 126 sequences.

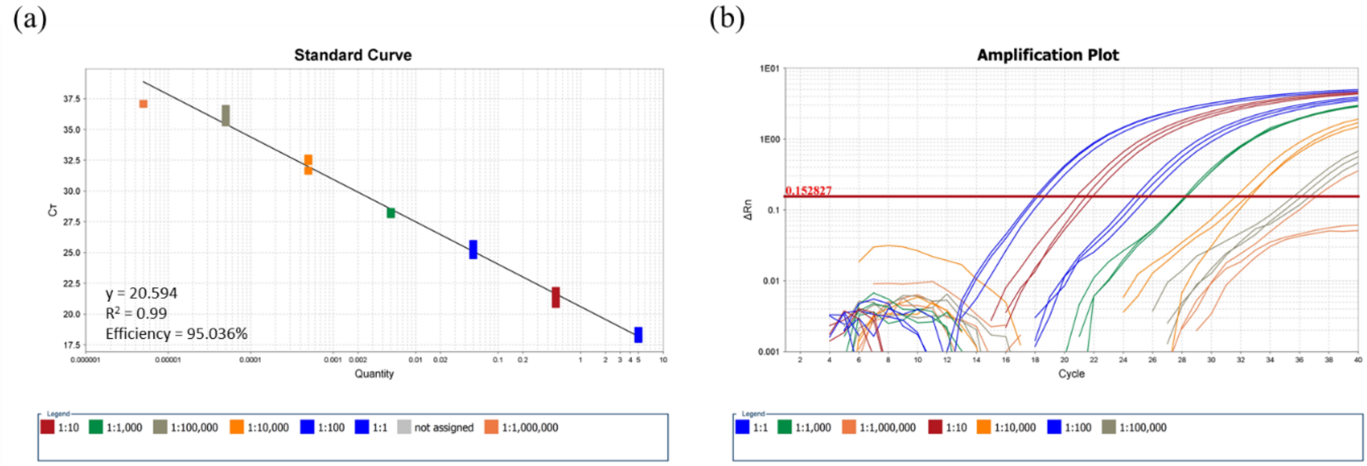

Supplementary Figure S2. Relative grapevine fleck virus (GFkV) quantification of the GFkV-CP assay. (**a**) Amplification plot and (**b**) Standard curve. Cycle quantification (Cq) values obtained for three replicates of ten-fold serial dilutions of GFkV control are plotted.
